# Supplementary material for: Functional Status of Patients over 65 Years Old Intervened on for a Hip Fracture One Year after the Operation
Source: Healthcare (Basel). 2023 May 22;11(10):1520. doi: 10.3390/healthcare11101520 (PMC10217831; doi:10.3390/healthcare11101520)
Supplement: Supplementary file 1 [file healthcare-11-01520-s001.zip › healthcare-2388198-supplementary.pdf]

Supplementary material.

Figure S1. Flow chart.

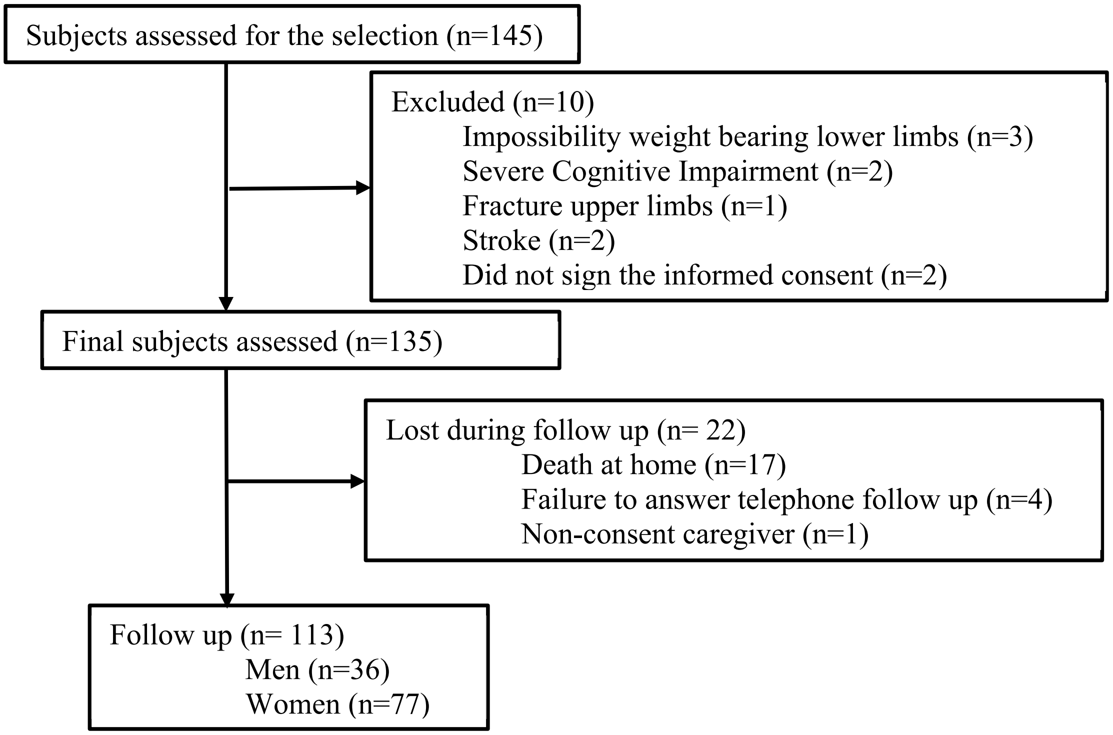

Table S1. Information on the total number of patients considered at admission.

	Sex			
	Female (N = 97)	Male (N = 38)	p-value	Total (N = 135)
Age, years	82.2 ± 7.4	80.2 ± 8.0	0.170	81.6 ± 7.6
Body mass index, kg/m^2	26.7 ± 4.5	27.0 ± 4.1	0.760	26.8 ± 4.4
Type of fracture, n (%)			0.878	
Intracapsular	40 (41)	17 (45)		57 (42)
Subtrochanteric	13 (13)	4 (10)		17 (13)
Pertrochanteric	44 (46)	17 (45)		61 (45)
Osteosynthesis material, n(%)			0.381	
Nail	61 (63)	21 (55)		82 (61)
Partial prosthesis	30 (31)	16 (42)		46 (34)
Total prosthesis	6 (6)	1 (3)		7 (5)
Charlson index	4.7 ± 1.5	4.9 ± 1.6	0.593	4.8 ± 1.5
Delirium	10 (10)	-	0.062	10 (7)
Number of physiotherapy sessions	5.2 ± 3.1	6.0 ± 3.6	0.171	5.4 ± 3.3
Days of hospital stay	13.3 ± 7.1	15.8 ± 8.5	0.087	14.0 ± 7.6
Patient location before, n (%)			0.269	
Home	92 (95)	34 (90)		126 (93)
Elderly residence	5 (5)	4 (10)		9 (7)
Patient location after, n (%)			0.827	
Home	81 (84)	31 (82)		112 (84)
Social health centre	8 (8)	3 (8)		11 (8)
Elderly residence	7 (7)	4 (10)		11 (8)
Functional tests				
Katz				
Average ± s.d.	4.4 ± 1.2	4.8 ± 0.7	0.015	4.5 ± 1.1
Katz, n (%)			0.127	
Severe disability (0-2)	8 (8)	1 (3)		9 (7)
Moderate disability (3-4)	24 (25)	5 (13)		29 (21)
Mild or no disability (5-6)	65 (67)	32 (84)		97 (72)
Lawton				
Average ± s.d.	4.4 ± 2.7	5.7 ± 2.5	0.012	4.8 ± 2.7
Median (P_{25}; P_{75})	5 (2; 7)	6.4 (4.4; 8)	0.008	5 (2; 8)
Lawton, n (%)			0.227	
Severe dependence (0-2)	29 (30)	6 (16)		35 (26)
Mild dependence (3-5)	28 (29)	12 (32)		40 (30)
Independence (6-8)	40 (41)	20 (53)		60 (44)
FAC				
Average ± s.d.	4.2 ± 0.9	4.4 ± 0.6	0.086	4.2 ± 0.8
FAC, n (%)			0.210	
Walking with aid (0-1)	2 (2)	-		2 (1)
Supervised walking (2-3)	14 (14)	2 (5)		16 (12)
Independent walking (4-5)	81 (84)	36 (95)		117 (87)
Sarcopenia				
Sarc-F				
Average ± s.d.	3.3 ± 2.5	2.0 ± 1.9	0.004	2.9 ± 2.4
Median (P_{25}; P_{75})	3 (1; 5)	1 (0; 3)	0.005	3 (1; 4)
Sarc-F ≥ 4, n (%)	40 (41)	8 (21)	0.030	48 (36)
Cognitive Impairment				

Pfeiffer				
Average ± s.d.	4.0 ± 2.6	3.6 ± 2.4	0.428	3.9 ± 2.5
Median (P_{25}; P_{75})	4 (2; 6)	4 (1; 6)	0.527	4 (2; 6)
Pfeiffer, n (%)			0.451	
No impairment (0-2)	30 (31)	15 (39)		45 (33)
Mild impairment (3-4)	25 (26)	8 (21)		33 (24)
Moderate impairment (5-7)	30 (31)	14 (37)		44 (33)
Severe impairment (8-10)	12 (12)	1 (3)		13 (10)

s.d. = standard deviation. P_{25}; P_{75} = Percentile 25 and 75, respectively. n=number of cases. FAC=Functional Ambulation Classification. Katz index=basic daily life activity. Lawton and Brody scale=instrumental activity daily life. Pfeiffer = Quantitative cognitive function.

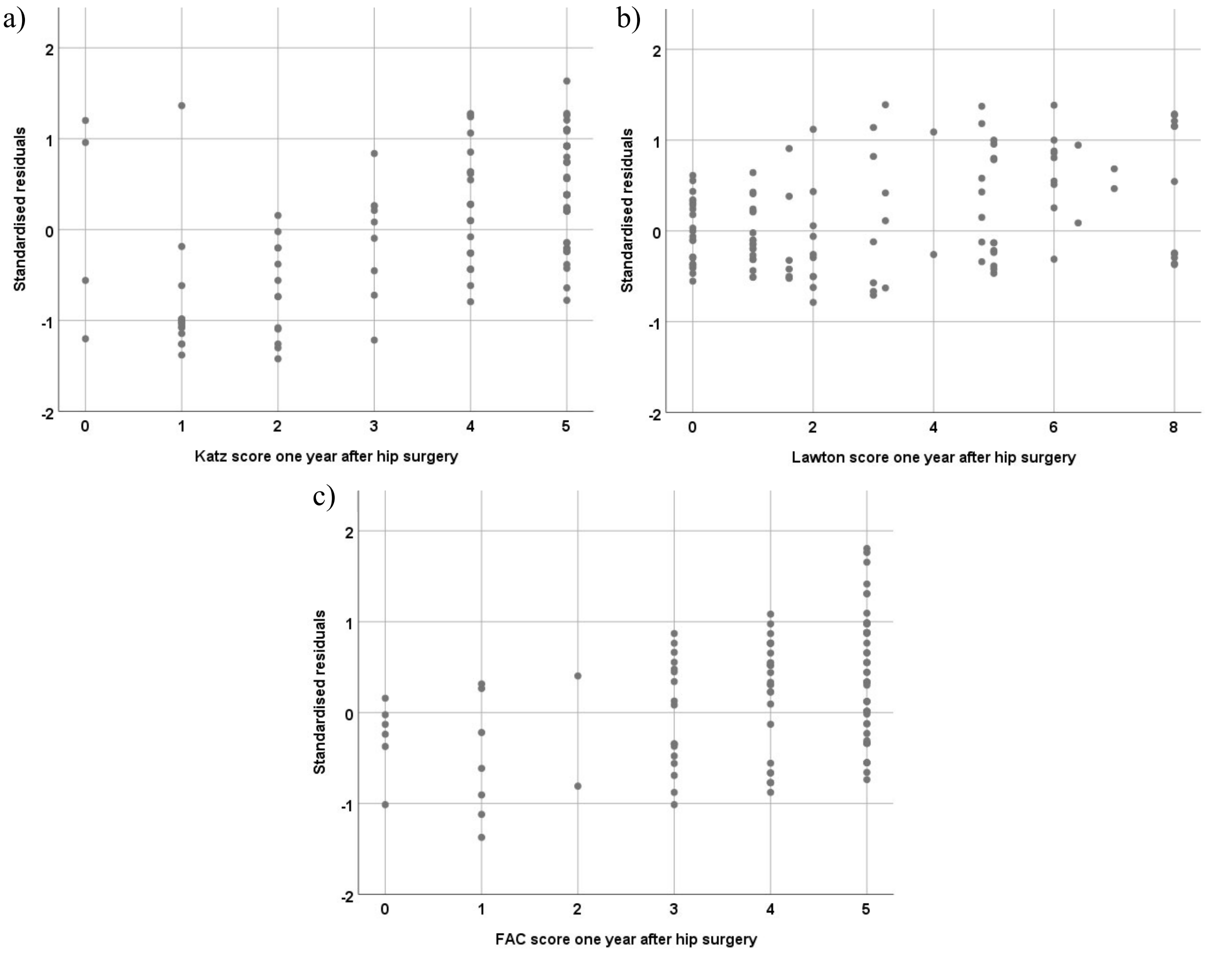

Figure S2. Standardized residuals of the models in Table 2 versus the values observed one year after the hip operation of the scores of (**a**) Katz, (**b**) Lawton and Brody, and (**c**) FAC.
